# Topics of Public Interest

**Published:** 1912-06

**Authors:** 


### TOPICS OF PUBLIC INTEREST

St. Paul now boasts of a 12-story office building exclusively for physicians and dentists. It is a block long, 50 feet deep, 200 feet high, with laboratory and emergency operating room on the roof. The building is fire-proof, sterilizable and has all sorts of devices for the safety and convenience of its occupants. The cost was a million.

Think of all that expense and bother, when all that is necessary is an old family mansion, with a new coat of paint, a little plaster of Paris in the rat holes, and a few partitions.

---

Ithaca has just lost a $50,000 school building by fire. 500 children just escaped with their lives, not even having time to get their wraps. Last winter, the high school burned. Buffalo has recenlty lost the Masten Park High School. Fire drills, efficient discipline and cool headedness of teachers and pupils have prevented loss of life in these three recent large fires of school buildings. But the BUFFALO MEDICAL JOURNAL sticks to its contention that every school house of any considerable size, built after today, should be as nearly fire-proof in its construction and equipment as is at all possible—not simply because of the direct danger, but because fire-proof construction and equipment imply approximate germ-proof environment and because both as a protection against fire and bacteria, the school house if properly built would be a constant object lesson to every pupil in attendance.

---

### Tobacco Facts

HERE are some facts on tobacco in the United States, taken from the internal revenue reports from 1911:

Output of large cigars in 1911, 7,270,144,822; increase, 200,-336,076.

Little cigars in 1911, 1,207,748,118; increase, 160,010,239.

Cigarettes in 1911, 9,828,682,005; increase, 1,184,124,915.

Manufactured tobaccò, pounds, 389,865,917; decrease, 54,-275,433.

---

THE so-called West Farm site has been secured for a Tuberculosis Hospital for Buffalo, at a cost of $650,000.

---

EXAMINATION FOR ARMY MEDICAL CORPS, will be held July 15 and September 3, at various points which will be selected so far as possible, to accommodate the majority of applicants. Full information may be secured on application to the Surgeon General, U. S. A., Washington, D. C. Without anticipating such information, we may say that there is no use for any one over 30 to apply, nor for one who has not had a year's hospital experience, nor for anyone who has not at least a fair general education, nor for anyone who cannot pass somewhere near the upper ten per cent. of a class. Nor do we believe that it is worth while to seek appointment unless with the idea of making a life work of it. The pay is good, much less than may be made in private practice, much more than most physicians do make; the work is interesting and not too hard; ample vacations are allowed and, while one is theoretically under orders, practically he is much more independent than in private practice. 68 vacancies exist, about enough for 1½ per cent. of the graduates of last year.

---

In the decade 1901-1910, the general death rate of the U. S. fell from 1655 to 1495.8 per 100,000 living. Meantime, the tubercular mortality fell from 196.9 to 160.3. It may be considered certain that the tuberculosis death rate is declining, both relatively to population, and to the total death rate. And we may add, as a personal opinion, that it is phthisiophobia that has done it, not that mere fear of the disease could have availed anything, nor with disregard for the trend of education, but in the sense that the people would not have been taught if they had not feared and avoided the disease.

---

LATE in April, a denist of Smethport, Pa., treated an ulcerated tooth for a patient who, next day, was discovered to have small pox. The dentist broke quarantine and the police of several places are searching for him. We have inquired as to the result but the local Police Headquarters are unable to give any information.

---

STERILIZATION OF CRIMINALS. Certiorari proceedings have been brought to test the constitutionality of the recently passed New Jersey law, the first victim having been selected for operation. The usual contention that the law is "cruel, inhuman and unusual" will be made. A similar law has just been signed in New York, six states in all now providing for sterilization.

---

METHYL ALCOHOL POISONING. What at first appeared to be botulysmus, among a number of inmates of a night shelter in Berlin, has turned out to be wood alcohol poisoning. 89 deaths and 5 cases of total blindness have resulted. The man who prepared the beverage has been sentenced to five years' imprisonment—which rather upsets our ideas as to the rigor of German justice.

---

REPORTING TUBERCULOSIS. In the last four years, 32 deaths from tuberculosis occurred in Tonawanda, one previously reported to the board of health; in N. Tonawanda, 41 deaths, 11

previously reported. Dr. Charles S. Prest of the State Department of Health has recently been in the Twin Cities, asking disagreeable questions as to why the law has not been obeyed. He expects to return June 3, to be still more disagreeable to any physicians who persist in violating the law. It was just on account of such occurrences, and to save our readers trouble, that we secured the very valuable summary of the laws and ordinances, from Dr. Fronczak. Our columns are the proper place to thresh out questions as to the advisability, rationality, efficiency and improvement of health laws.

---

THE University of Buffalo announces the following faculty changes:

Dr. F. C. Busch has resigned, as Professor of Physiology and will be succeeded by Dr. Frederick H. Pratt who is, at present, teaching this subject at Wellesley College and who was formerly assistant in physiology at the Harvard Medical School. Dr. Pratt enters upon a position of distinction. The experimental teaching of Austin Flint, the founder of the BUFFALO MEDICAL JOURNAL, was pioneer work not only for the University of Buffalo but for the medical world. Many men in active practice today remember the lucid and entertaining lectures of Julius Pohlman who, more than the great majority of teachers in any line of thought, had the knack of drawing practical lessons from theoretic science and of impressing his points so that they remained permanent in the mind of the pupil. With Dr. Busch, was inaugurated the standard of more recondite methods in a branch which, according to the older custom was regarded by students as of minor importance and therefore, justly demanding a small share of their attention and effort. Dr. Busch leaves the didactic field in order to devote his time to the clinical work of the N. Y. State Cancer Hospital and Dr. Pratt's assumption of service marks a further step in medical education, namely the recognition of a distinct didactic profession. While on many accounts, the association of students with teachers who were also men in active practice, has been and is an advantage and probably must continue for certain subjects, the relegation of medical teaching to teachers has a great advantage on the didactic side and also obviates the friction, lack of harmony and ethical restlessness of the profession to which the joint assumption of practice and teaching has unfortunately led to in many instances.

Dr. DeWitt H. Sherman succeeds Dr. Eli H. Long as Professor of Materia Medica and Therapeutics. It is a logical promotion, inevitably calling forth the regret that time is passing, yet the regret is mitigated by the fact that the change long anticipates any necessary or established age period. Rather let us take pleasure in complimenting both men on excellent work, and in extending good wishes for future activities.

As was expected, the recent appointment of Dr. Herbert Upham Williams as Acting Dean, has been made permanent.

---

Prevalence of Typhoid in Buffalo.—The warning to boil the city water has, possibly, given an erroneous idea that an epidemic exists. As a matter of fact, typhoid has been less prevalent than usual except that, with the late breaking up of the ice in the lake and the agitation of the water, there has been the usual increase but thrown into May instead of an earlier month. The figures are as follows (courtesy of Dr. Gram.)

| 1911 | | 1912 | |
|---|---|---|---|
| 33 | January | 26 | |
| 56 | February | 16 | |
| 64 | March | 10 | |
| 33 | April | 20 | |
| 33 | May | 39 | up to May 18 |
| 219 | | 111 | |

---

The Buffalo Association for the Relief and Control of Tuberculosis, at its recent annual meeting, urged the erection of a special children's hospital on the Perrysburg site. In view of the present high tax rate of Buffalo, it was suggested that the amount necessary, $10,000 for a temporary structure, be raised by popular subscription. Mr. Irving S. Underhill was elected President.

---

The Poster Advertising Association, comprising 3,500 firms throughout the United States, has decided to refuse patent medicine advertisements and will endeavor to eliminate objectionable theatric advertisements. We have previously voiced the optimistic sentiment that, in the long run, the medical profession can depend upon the support of business men generally, for any movement in the interest of the community, provided that we do not inject too much professional "ethics" into it.

---

The Board of Health of Corning has authorized the employment of a visiting nurse to care for and to discover tuberculous cases.

THROUGH the generosity of Mr. Arnold Gregory, a large and well located residence has been bought in order to provide Albion with a hospital. As the Benedict and the Gregory boys began marrying each other's sisters about as soon as they landed in this country, we feel almost a personal, at least a family interest in Mr. Gregory's gift.

---

THE following state civil service examinations, to be held June 15, may interest physicians. For detailed information and application blanks, address the State Civil Service Commission at Albany. Inspector of Vocational Schools, male, $2,500. Medical Interne, state hospital service, $600 and maintenance. Health Officer and Inspector, Canandaigua, $400.

---

### New Inventions

Frank Kuhn, Detroit, Mich. Assignor to Amer. Electrical Heater Co., Detroit, Mich. 1,025,144. Electric Footwarmer.

F. J. Jaubert, Paris, France. 1,025,191. Cartridge for the Preparation of Oxygen.

H. W. Sanford, Washington, D. C. 1,025,207. Surgical Kit.

E. M. Grindle, Holton, Kas. 1,025,265. Cheek-Distender.

Jas. Birrell and Wm. Birrell, Seattle, Wash. Assignors to Birrell Vacuum Vibrator Co., Seattle, Wash. 1,025,504. Combined Vibrator and Vacuum Apparatus.

C. A. Howe, Chicago, Ill. 1,025,571. Foot-Support.

T. J. Donovan, Syracuse, N. Y. 1,025,612. Bathing Machine.

R. M. Machlett, New York, N. Y. 1,025,635. Gas-Regulator for Gas-Tubes.

D. M. Don Jian, Washington, D. C. 1,025,903. Device for Removing and Curing Corns.

J. F. Craven, Pittsburgh, Pa. Assignor to Craven Engineering Co., Pittsburgh, Pa. Receptacle for containing and discharging semisolid and pasty substances. 1,025,511, 1,025,512 and 1,025,513.

Franz Elger, Basel, Switz., assignor to Hoffman-La Roche Chemical Works, New York, N. Y. Process of Separating Meta- and Para-Cresols. 1,025,616.

G. F. Jaubert, Paris, France. Cartridge for the preparation of Oxygen. 1,025,191.

Otto Schmidt, Mannheim, Ger., assignor to Badische, Anilin & Soda Fabrik, Ludwigshafer-on-the-Rhine, Ger. Chlor-Aralykl-Sulfonic acids and process of making such compounds. 1,025,652.

Heinrich Hoerlein, Vohuxnkel, near Elberfeld, Ger., assignor to Farbenfabriken vorm. Friedr. Bayer & Co. Phenylethylbarbituric Acid. 1,025,872.

Nathan Sulzberger, New York, N. Y. Chloral Derivatives containing the Radical of a Fatty acid. 1,025,889.

Otto Liebknecht Frankfort-on-the-Main. Ger., assignor to Roessler & Hasslacher Chemical Co., New York, N. Y. Stable Hydrogen-Peroxid Solution. 1,025,948.

1,023,803. Sucking Bottle. Chas. DeBock, Lele, Belgium.

1,023,822. Teething Finger. Arthur Dubay, Gardiner, Me.

1,024,152. Method and Means for Producing Dental Plates. Henry J. Smith, Phila, Pa.

1,024,153. Dental-Plate. Henry J. Smith, Phila, Pa.

1,024,122. Medical Spraying Syringe, Moritz Saenger, Madgeburg, Germany.

H. J. Morris & J. A. Cowlyn, Kansas City, Mo. Artificial Limb. 1,026,109.

H. M. Crawford, Lima, Ohio. Electric Water Heating Syringe. 1,026,564.

W. H. Herman, Wesson, Miss. Bottle-Vending Machine, 1,025,978.

Robert Kahn, Frankfort-on-the Main, Ger. Assignor to Farbwerke Vorm, Meister Lucius & Bruning Hochst-on-the-Main, Ger. Arseno Compounds and process of making same, 1,026,094.

O. C. Schultz, Chicago, Ill. Assignor to Bauer & Black, Chicago, Ill. Machine for making Surgical Bandages, 1,026,283.

Fred Bedford and Chas. Edw. Williams, Sleaford, Eng. Process for the conversion of Unsaturated Fatty Acids, their Glycerids and Esters into the corresponding saturated Compounds, 1,026,339.

M. L. Rusk, New York, N. Y. Water-Bottle for Infants, 1,026,460.

J. D. Wise, Jackson, Tenn. Medicine-Dropper, 1,026,541.

M. H. Shoenberg, San Francisco, Cal. Assignor to The Presto Electrical Mfg. Co., San Francisco, Cal. Electrically-Heated Syringe. 1,026,611.

Donald McKay, Collingwood, Ont., Can. Truss. 1,026,758.

J. K. Toles, Berkeley, Cal. Combined Container and Cutter. 1,026,778.

Wilhelm Hiemenz and Walter Kropp, Elberfeld, Ger. Assignors to Frabenfabriken Vorm Friedr. Bayer & Co., Elberfeld, Ger. Pharmaceutical Product. 1,026,913.

